# Observation of carrier localization in cubic crystalline Ge_2_Sb_2_Te_5_ by field effect measurement

**DOI:** 10.1038/s41598-017-18964-w

**Published:** 2018-01-11

**Authors:** Hang Qian, Hao Tong, Ming-Ze He, Hong-Kai Ji, Ling-Jun Zhou, Ming Xu, Xiang-Shui Miao

**Affiliations:** 1 Wuhan National Laboratory for Optoelectronics (WNLO), Huazhong University of Science and Technology (HUST), Wuhan, 430074 China; 2 School of Optical and Electronic Information, Huazhong University of Science and Technology, Wuhan, 430074 China

## Abstract

The tunable disorder of vacancies upon annealing is an important character of crystalline phase-change material Ge_2_Sb_2_Te_5_ (GST). A variety of resistance states caused by different degrees of disorder can lead to the development of multilevel memory devices, which could bring a revolution to the memory industry by significantly increasing the storage density and inspiring the neuromorphic computing. This work focuses on the study of disorder-induced carrier localization which could result in multiple resistance levels of crystalline GST. To analyze the effect of carrier localization on multiple resistant levels, the intrinsic field effect (the change in surface conductance with an applied transverse electric field) of crystalline GST was measured, in which GST films were annealed at different temperatures. The field effect measurement is an important complement to conventional transport measurement techniques. The field effect mobility was acquired and showed temperature activation, a hallmark of carrier localization. Based on the relationship between field effect mobility and annealing temperature, we demonstrate that the annealing shifts the mobility edge towards the valence-band edge, delocalizing more carriers. The insight of carrier transport in multilevel crystalline states is of fundamental relevance for the development of multilevel phase change data storage.

## Introduction

Phase change memory (PCM) is one of the most important candidates for future nonvolatile memory devices owing to its high scalability, high speed, and long endurance^1^. Chalcogenides adopted in PCM can be switched between a high-resistive amorphous (RESET) and a low-resistive crystalline (SET) state via different current pulses^2,3^. The main challenge for commercial applications of PCM is to reduce the power consumption because the RESET process requires high current density to melt the crystalline region. The prototypical PCM material Ge_2_Sb_2_Te_5_ (GST) has been intensively studied in the past a few years^4–6^. A lot of work have been done for the understanding of carrier transport due to its great importance for the crystalline state of PCM^7^. It has been reported that metal-insulator transition (MIT) upon annealing can be observed in crystalline GeSbTe compounds, and the Anderson localization resulted from the vacancy disorder is responsible for this MIT^8–10^. The control of the degree of disorder in crystalline state is important in achieving multilevel resistance states, and thus the study of the disorder-induced localization is necessary. Disorder-induced carrier localization has been investigated by low temperature transport experiments, and its origin has been revealed by density functional theory simulations^11^. The vacancy ordering process was also demonstrated by high angle annular dark field scanning transmission electron microscopy (HAADF-STEM) experiments^12,13^. Previous works based on the Hall effect and conductivity measurement have limitation for that the Hall effect is not expected to yield the exact number of carriers once they are localized^14,15^. Besides, a small fraction of non-stoichiometric vacancies lead to a large concentration of carriers on the order of 10^20^ cm^−3^ ^16,17^, which hampers the conductivity analysis^10,18,19^. Due to its close relationship with carrier transport, the analysis of field effect (the intrinsic effect of a transverse electric field on surface conductance) has been well practiced in MIT mechanisms including electron correlation (Mott transition)^20,21^ and disorder (Anderson localization)^22,23^. Field effect studies of semiconductors have been a very active area of interest for many years, because they can probe the localized states of carriers which control the electronic properties of materials^24–26^. However, carrier transport of crystalline GST observed from field effect has not been reported before. In this work, we designed a back-gated structure to measure the field effect of GST in cubic crystalline state. We analyzed temperature dependence of the field effect mobility of crystalline GST and the delocalization of carriers as the annealing temperature (*T*_*a*_) increases. This study provides observation of localized carriers in different crystalline states of GST and thus enriches the research of MIT in the GeSbTe compounds as an addition to conventional transport measurement techniques. Insights of carrier transport in GeSbTe compounds have implications for the development of low power consumption and multilevel memory devices.

## Results

**Figure 1:**
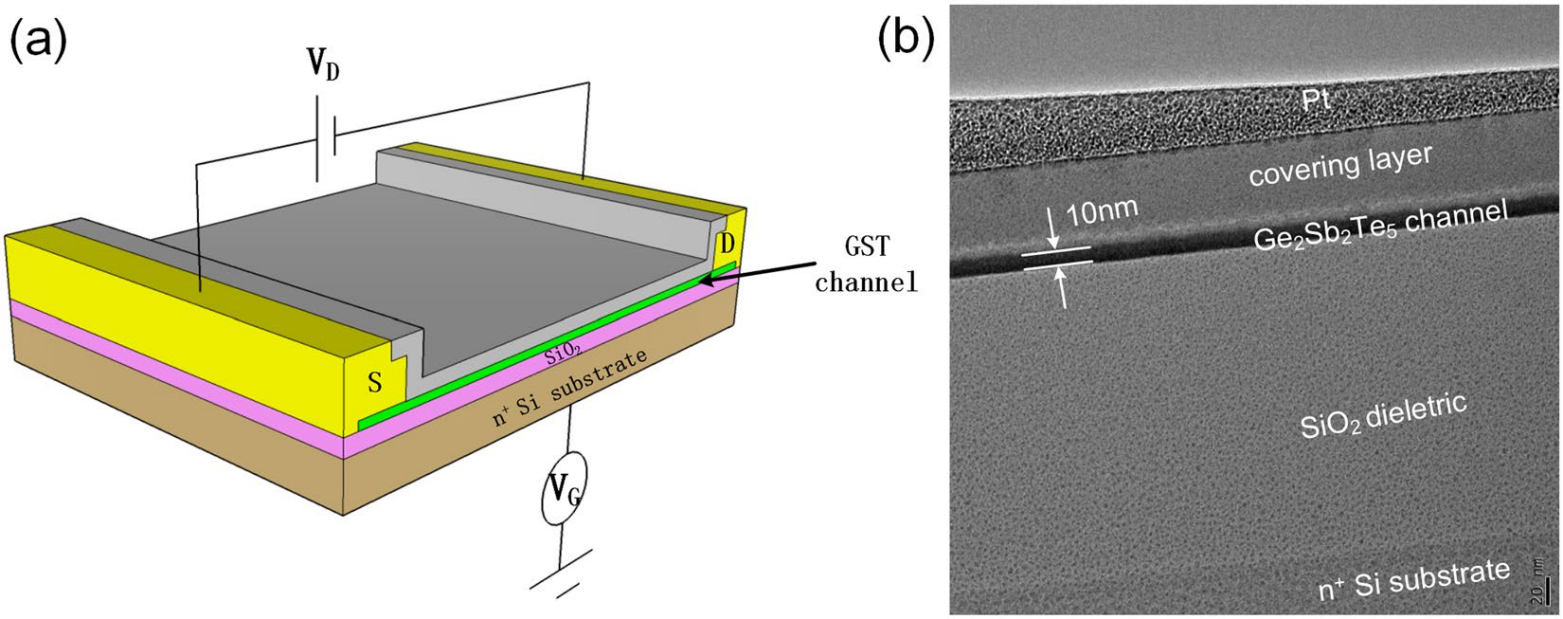
(**a**) Sketch of the device structure. When a gate voltage (*V*_*G*_) is applied, carriers are induced in the semiconductor. The drain voltage (*V*_*D*_) is applied between source and drain electrodes, and the resulting channel current (*I*_*D*_) was monitored to detect the field effect. (**b**) TEM image of the back-gated structure device. The thickness of GST film is controlled to 10 nm.

Figure 1(a) depicts the back-gated structure of our device. The device consists of two parallel electrodes (source and drain) and a gate electrode which is isolated from the GST channel by an insulating layer. The gate, the insulator, and the semiconductor form a parallel-plate capacitor. When a gate voltage (*V*_*G*_) is applied on this capacitor, carriers are motivated in the semiconductor. The drain voltage (*V*_*D*_) is applied between source and drain electrodes, and the resulting channel current (*I*_*D*_) is monitored to detect the field effect. The effect of the electric field is limited to a channel thickness within nanometers, owing to the presence of Thomas-Fermi screening^27^. The noise of channel current is comparable to or even stronger than the field effect induced current (*I*_*D*_) when it is high and thus, the thickness of GST film is controlled to 10 nm so that the noise is reduced and Δ*I*_*D*_ is revealing. As shown in Fig. 1(b), transmission electron microscopy (TEM) measurement was performed to characterize the structure of our device. The insulating dielectric layer is 200 nm, and such large thickness as well as the high quality of films ensures low leak current when the high voltage is applied on the back gate. The GST channel was fabricated accurately to 10 nm with high uniformity. Details of TEM and material composition analyzed by energy dispersive X-ray Spectrum (EDS) are described in the Figure S1 of Supplemental materials.

**Figure 2:**
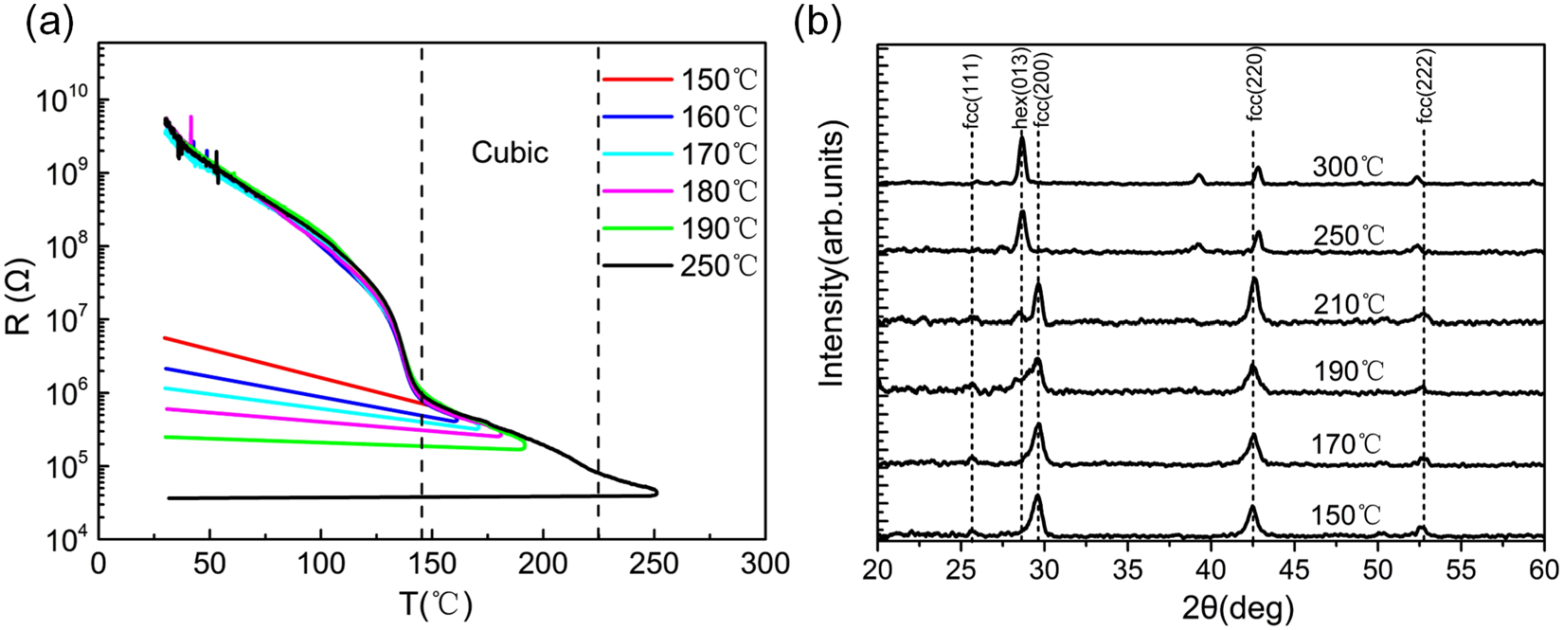
(**a**) Temperature dependence of the GST devices resistance with step annealing temperature. The thermal annealing induced MIT is observed. (**b**) Evolution of XRD patterns of 10 nm GST films with increasing *T*_*a*_. The GST has a fcc structure when the *T*_*a*_ is between 150 °C and 200 °C and transforms to the hexagonal phase above 250 °C.

We preformed the resistance measurements depending on annealing temperature of GST devices by thermal annealing *in-situ*. Starting at room temperature, the heating speed was kept at a constant rate of 5 Kmin^−1^. As shown in Fig. 2(a), the resistance of the device decreases smoothly at the beginning because the GST films are amorphous (which have an Arrhenius-like temperature dependence of the resistance). When the *T*_*a*_ reaches 140 °C, the resistance of devices drops abruptly by several orders of magnitude due to the crystallization of phase change materials. On further annealing to 250 °C, the resistance of the crystalline GST reduces again by almost two orders of magnitude. As has been demonstrated by Siegrist *et al*. using the samples of Ge_1_Sb_2_Te_4_^8^, this annealing effect in the electrical resistance of crystalline state takes place prior to the MIT transition when GST transforms into the hexagonal phase.

Our work focuses on the rather insulating samples because the carrier localization is expected to affect significantly the electrical transport. Those devices annealed between 150 °C and 190 °C were selected to carry out further field effect measurements. When the *T*_*a*_ exceeds 190 °C, the field effect of devices becomes mixed up due to the high conductance (The noise of *I*_*D*_ will cover up Δ*I*_*D*_ and measurement cannot be completed at the technical level). We stabilized the samples at the target *T*_*a*_ for one more hour to guarantee that the samples are fully crystallized. XRD measurements were performed to obtain detailed structural information about films. The GST films measured by XRD were fabricated with the same procedure as those in back gate devices, i.e., they were annealed under the same condition as the devices. Figure 2(b) presents the evolution of XRD patterns of films with increasing *T*_*a*_. The XRD results of those films show that GST has a fcc structure when the *T*_*a*_ is between 150 °C and 200 °C. The peaks attributed to the cubic structure have been identified and indexed. The 2*θ* position of the (200) peak, which is the most intensive^28^, shifts from 29.2° to 28.3° above the 250 °C due to the phase transformation from cubic to hexagonal phase. From the XRD measurements, we further confirm that the GST films used in field effect measurements exhibit cubic structure.

**Figure 3:**
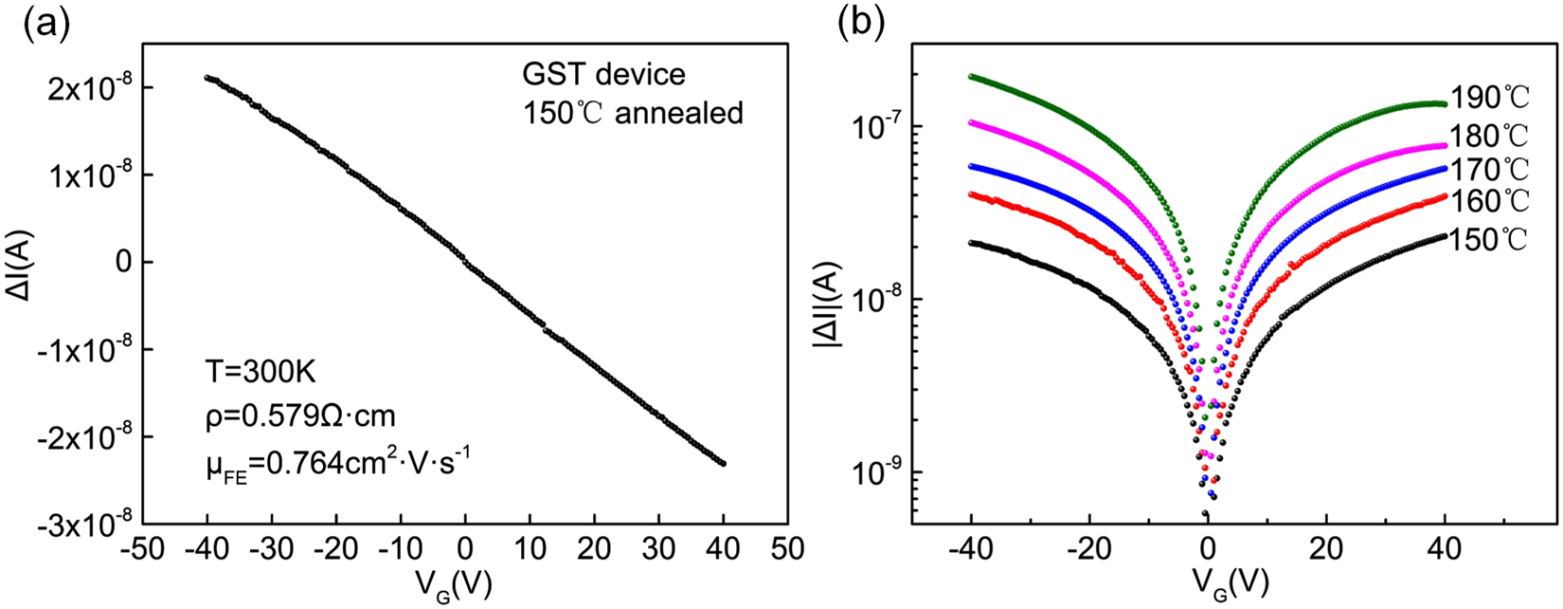
(**a**) Gate modulation of drain current for GST sample annealed at 150 °C (**b**) Change in source-to-drain current versus gate voltage with increasing *T*_*a*_. The field effect induced current increases significantly with the increasing *T*_*a*_.

Field effect measurements are shown in Fig. 3 for GST samples as the current change in channel with *V*_*G*_. A small bias was applied between the source and drain electrodes, and the resulting change in the conductance was measured. As shown in Fig. 3(a), the relationship between Δ*I* and *V*_*G*_ in sample with 150 °C annealing is described as an example which is expected to have highest degree of disorder in all samples. The output characteristics are completely in the linear regime. We further performed field effect measurements on the devices with increasing *T*_*a*_ to analyze the effect of annealing on the carrier localization. The drain current of all the evaluated device types shows a small and approximately linear dependence on *V*_*G*_, which indicates that the mobility of induced carriers is independent of *V*_*G*_. More details of each sample type have been described in the Figure S2 of Supplementary materials. The field effect measurement of devices with *T*_*a*_ between 150 °C and 190 °C have been performed. As shown in Fig. 3(b), the field effect induced current increases significantly with the increasing *T*_*a*_.

## Discussion

The metastable crystalline GST has a rocksalt-like structure containing the vacancy fraction of around 20% (varying with compositions in GeSbTe compounds) on the cation sublattice. Te atoms occupy one fcc sublattice, while Ge and Sb atoms as well as vacancies occupy the other sublattice randomly^29^. This structure feature inevitably leads to the vacancy disorder in the Ge/Sb sublattice^11^. From the step annealing process, we observed the annealing effect in the electrical resistivity of crystalline state, which is consistent with the MIT demonstrated by previous research^8,9^. Our field effect analysis focuses on the insulating part of the MIT, and the XRD measurement confirms that all GST in devices used in the field effect measurements belong to the cubic system instead of a hexagonal one.

**Figure 4:**
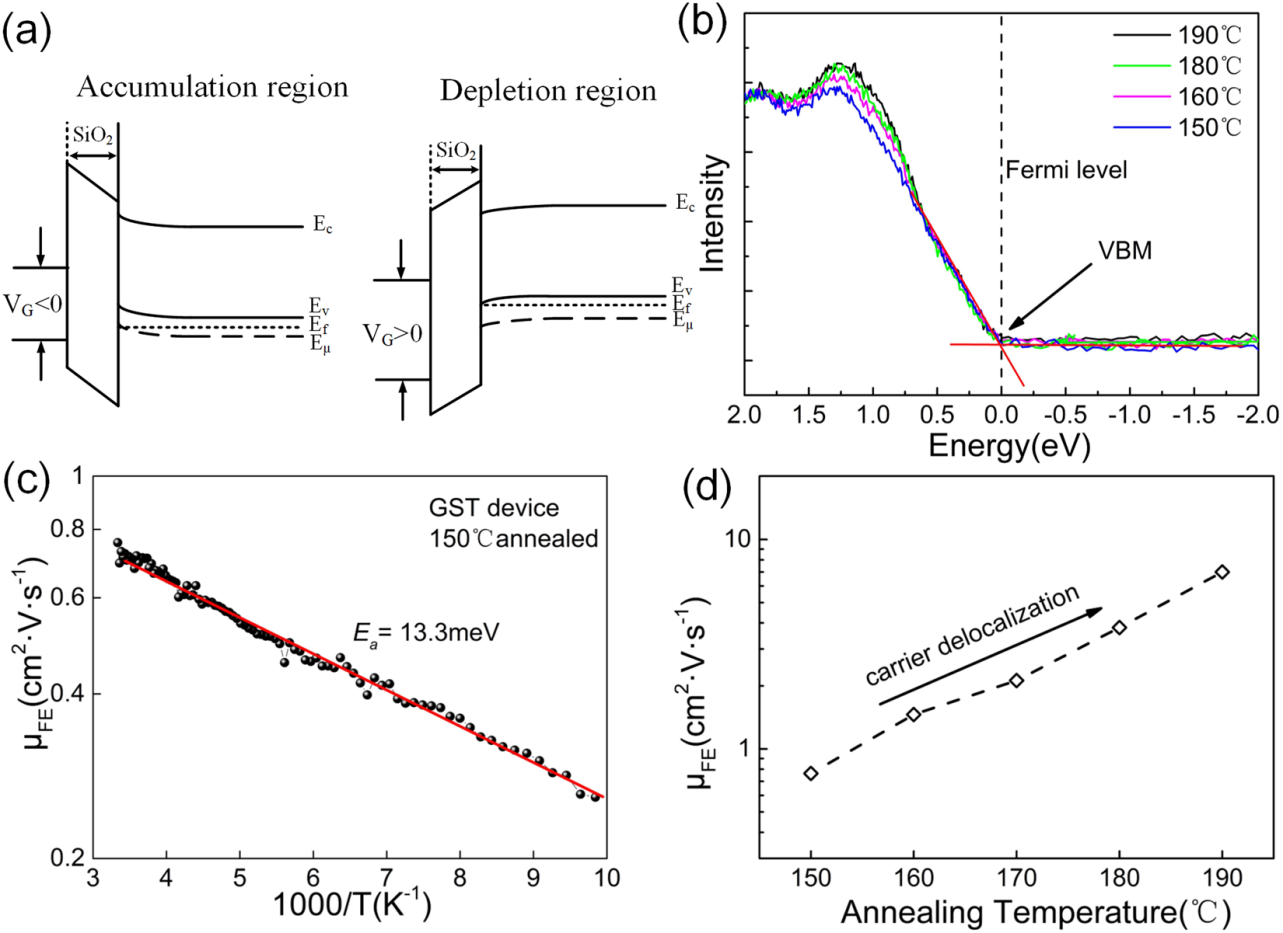
(**a**) Band diagram of MIS structure under applied bias voltage. (**b**) The XPS spectra of the valence band for GST films with increasing *T*_*a*_ between 150 °C and 190 °C. The Fermi level (E = 0 eV) of GST films are all located near the VBM. (**c**) Extracted excess charge field effect mobility against temperature showing the Arrhenius mobility-temperature relationship. (**d**) Field effect mobility versus annealing temperature. The field effect mobility increases with increasing annealing temperature, indicating the carrier delocalization.

We analyzed the carrier transport based on the field effect results, and find that the carriers induced in the channel are not located directly at the semiconductor-insulator interface, but are instead distributed spatially inside the bulk. This feature is probably responsible for the field effect. In field effect measurement, the energy bands near the surface is displaced relative to the Fermi level. The conductance of the sample is changed by such a displacement because the carrier concentrations in the space charge region are different from the bulk concentrations. In “depletion”, carriers are removed from the semiconductor volume, whereas in “accumulation” they are added. The “inversion” of transportation type will occur if a p-type semiconductor with a large positive charge on the surface causes an n-type surface layer^30^. Figure 4(a) shows the behavior of the energy bands for a metal-insulator-semiconductor (MIS) structure. Depletion characteristics are produced by positive gate voltages, and accumulation characteristics are produced by negative voltages. This is a convincing evidence that the electrical transport process in GST features essentially unipolar conduction by hole carriers. From all the available data, there is no detectable transition from depletion to inversion characteristics over accessible range of gate voltage. The crystalline GST has been generally considered as a degenerate semiconductor with narrow band gap of ~0.5 eV^31,32^. Figure 4(b) shows the X-ray photoemission spectra (XPS) recorded near the valence-band maximum (VBM) of the GST film with *T*_*a*_ between 150 °C and 190 °C. We confirm that the Fermi levels (E = 0 eV) of GST films used in field effect measurements are all located near the VBM, consistent with previous studies on phase change materials^33,34^.

The experiment observes the change in the mobile carrier component induced by an applied gate voltage *V*_*G*_. If the field induced carriers are restricted in a region much narrower than the insulator thickness *t*_*ox*_ then the total field induced carriers per unit area *Q* is given by

1 $$Q=\frac{{V}_{G}{\varepsilon }_{ox}}{{t}_{ox}}$$

where *ε*_*ox*_ is the dielectric permittivity of the insulator. The total carriers consist of two components,

2 $$Q={Q}_{F}+{Q}_{L}$$

where *Q*_*F*_ is the free carrier and *Q*_*L*_ is the localized carrier. If the distance between the source and drain electrodes is *L* and the width of the conducting path is *W*, then the change in conductance Δ*G* is

3 $${\rm{\Delta }}G={\mu }_{0}{Q}_{F}\frac{W}{L}$$

where *μ*_0_ is the mobility of the free carrier. Thus, for a constant applied source-to-drain voltage *V*_*D*_, the field induced current Δ*I* can be written as

4 $${\rm{\Delta }}I=({\rm{\Delta }}G){V}_{D}={\mu }_{0}{Q}_{F}\frac{W}{L}{V}_{D}=(\frac{{\mu }_{0}{Q}_{F}}{{Q}_{F}+{Q}_{L}})\frac{W}{L}\frac{{\varepsilon }_{ox}}{{t}_{ox}}{V}_{D}{V}_{G}$$

The quantity in parentheses in Equation (4) is the effective mobility which is measured in field effect experiments and we define it as field effect mobility *μ*_*FE*_. Transport in the materials studied in the present investigation takes place via carrier motion in the extended states beyond the mobility edge, so that the field effect mobility will be related to the free carrier mobility *μ*_0_, by the ratio of free carriers to total excess induced carriers.

5 $${\mu }_{FE}={\mu }_{0}\frac{{Q}_{F}}{{Q}_{F}+{Q}_{L}}\approx {\mu }_{0}\frac{{Q}_{F}}{{Q}_{L}}$$

where the approximation of Equation (5) is valid when *Q*_*F*_ is much smaller than *Q*_*L*_. For the *μ*_*FE*_ measured in GST films is significantly small, the main contribution of induced excess carrier is the localized carriers in the band tail.

We obtain the mobility *μ*_*FE*_ from the gate modulation of drain current. The relationship between the excess carrier mobility and temperature was analyzed. As shown in Fig. 4c, the field effect mobility exhibits thermally activated with small activation energies of 13.3 meV, from room temperature to 100 K. Below 100 K the signal-to-noise ratio becomes too small for reliable measurements. The *μ*_*FE*_ shows the temperature activation, a hallmark of carrier localization^35^. Thus, we can further conclude that the field effect induced carriers are mainly localized.

As shown in Fig. 4(d), the field effect mobility was extracted and show positive correlation with the *T*_*a*_. The μ_0_ remains almost constant while the Fermi level changes little with the increase of annealing temperature^36^, which is proven in the XPS measurements as shown in Fig. 4(b). From the Equation (5), the increase of *μ*_*FE*_ is mainly due to the proportional reduction of localized carriers in all field induced carriers. We conclude that the annealing shifts the mobility edge towards the valence band edge, delocalizing more carriers^37^. We find evidence for that the mobility edge is approaching Femi level as the *T*_*a*_ increases.

In phase change materials, stoichiometric vacancies could result in disorder, whereas the carrier concentration is controlled by the excess vacancies^38^. It is hard to distinguish the contribution from disorder due to the high carrier concentration. In the field effect measurements, the change in conductance of semiconductor due to the space charge layer in which the extra carriers are induced. The important role of localized carrier in materials can be directly observed in spite of the high concentration of carriers. Through this method, we can confirm that the annealing shifts mobility edge on the insulating side of MIT, which has not been proven in previous work, to our knowledge. Hence, through gate induced current Δ*I*, the carrier localization in cubic GST was observed. The direct observation of localized carriers substantiates disorder induced localization in crystalline phase change materials. The field effect measurements demonstrate a new approach to explore facets of disorder-induced localization.

In summary, by field effect measurements, we investigated the disorder induced localization in cubic crystalline GST. We observed the linear area of field effect in GST back-gated devices and calculated field effect mobility. The temperature dependence of field effect mobility elucidates visually that the carriers are localized and the small activation energy was acquired. This work confirms that the annealing shifts the mobility edge towards the Fermi level, delocalizing more carriers. This method is effective in studying the disorder induced MIT in the presence of high carrier concentrations. The localized carriers are observed to determine the multilevel crystalline states of GST. These results shed light on the mechanism of the electric transport of multilevel states in crystalline GST, which provides conceptual guidance for the design of high-density memory devices.

## Methods

### Device fabrication

The devices were fabricated by thermally oxidizing a heavily n-doped crystalline silicon substrate to form a layer of SiO_2_ approximately 200 nm thick. UV-lithography was used to pattern the GST thin film channel and source/drain electrodes. The channel size was 10 μm × 100 μm. The GST film was deposited by DC magnetron sputtering from stoichiometric targets. The back ground pressure was kept smaller than 1 × 10^−6^ mbar and the process pressure was of the order of 5 × 10^−3^ mbar using argon as sputter gas. The thicknesses of the two layers were controlled via the deposition time and afterward by means of step height measuring instrument. The fabricated devices were capped with 50 nm ZnS:SiO_2_ (80:20) by RF sputtering, which can render the devices air stable.

### Microscopic analysis

The GST films used for XRD and XPS measurements were prepared at the same time and conditions as devices. The cross sectional samples for TEM were performed by the focused ion beam system (FIB). To minimize the beam damage during the FIB sample preparation, the multiple processes with reducing voltages and beam current are used. The composition of the films was confirmed as nearly Ge_2_Sb_2_Te_5_ by EDS.

### Electrical measurement

To obtain the field-effect modulation, the source was biased at 1 V and resulting drain current was monitored using Aglient B1500 semiconductor analyzer. The applied *V*_*G*_ can range up to ± 40 V, and all measurements were performed in the Ohmic region. The low temperature measurement was carried out in a Quantum Design Physical Property Measurement System (PPMS).

## Electronic supplementary material

Dataset1
