# Supplementary material for: Observation of carrier localization in cubic crystalline Ge2Sb2Te5 by field effect measurement
Source: Sci Rep. 2018 Jan 11;8:486. doi: 10.1038/s41598-017-18964-w (PMC5765150; doi:10.1038/s41598-017-18964-w)
Supplement: Supplementary file 1 — Dataset1 [file 41598_2017_18964_MOESM1_ESM.zip › Supplemental Materials.docx]

**Observation of carrier localization in cubic crystalline Ge_2_Sb_2_Te_5_ by field effect measurement**

Hang Qian^1,2^, Hao Tong^1,2*^, Ming-Ze He^1,2^, Hong-Kai Ji^1,2^, Ling-Jun Zhou^1,2^, Ming Xu^2^, and Xiang-Shui Miao,^1,2^

*^1^Wuhan National Laboratory for Optoelectronics (WNLO), Huazhong University of Science and Technology (HUST), Wuhan 430074, China*

*^2^**School of Optical and Electronic Information, Huazhong University of Science and Technology, Wuhan 430074, China*

*Author to whom correspondence should be addressed. Electronic mail: [tonghao@hust.edu.cn](mailto:tonghao@hust.edu.cn).


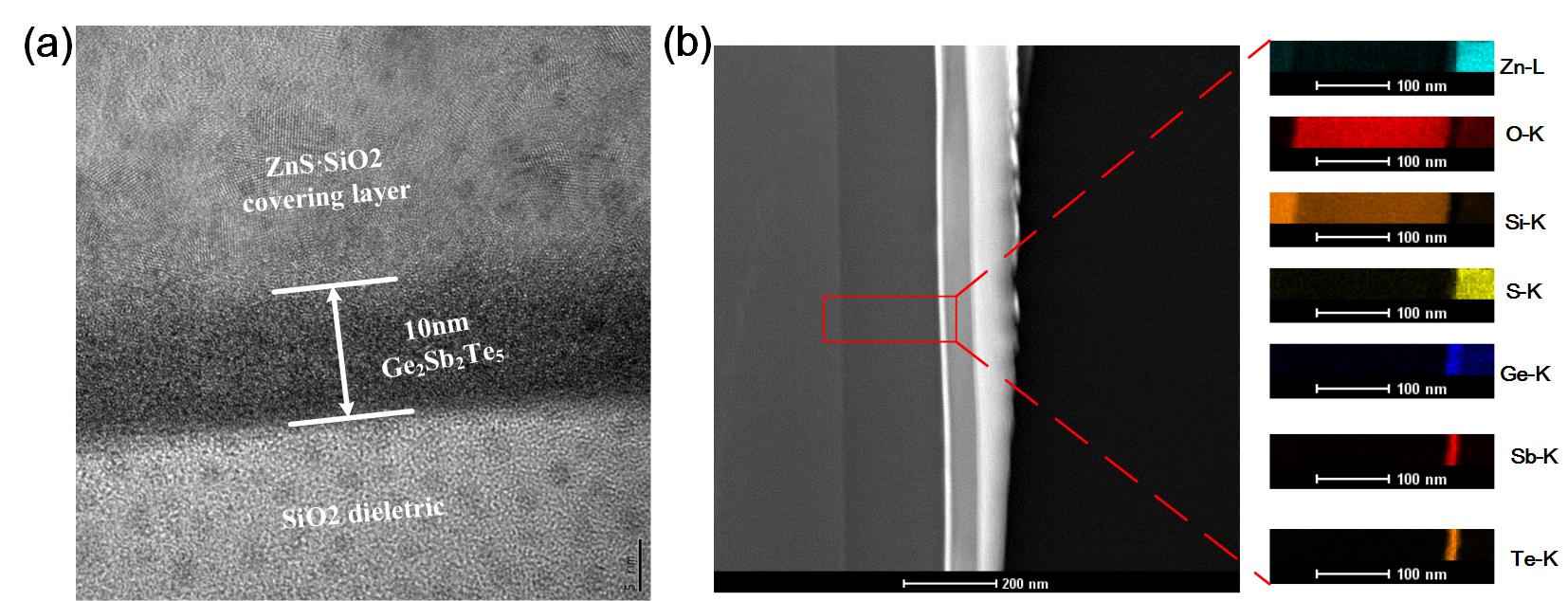


Figure S1. (a) TEM cross section of 10nm GST film in the devices. (b) EDS analysis of each layer of devices


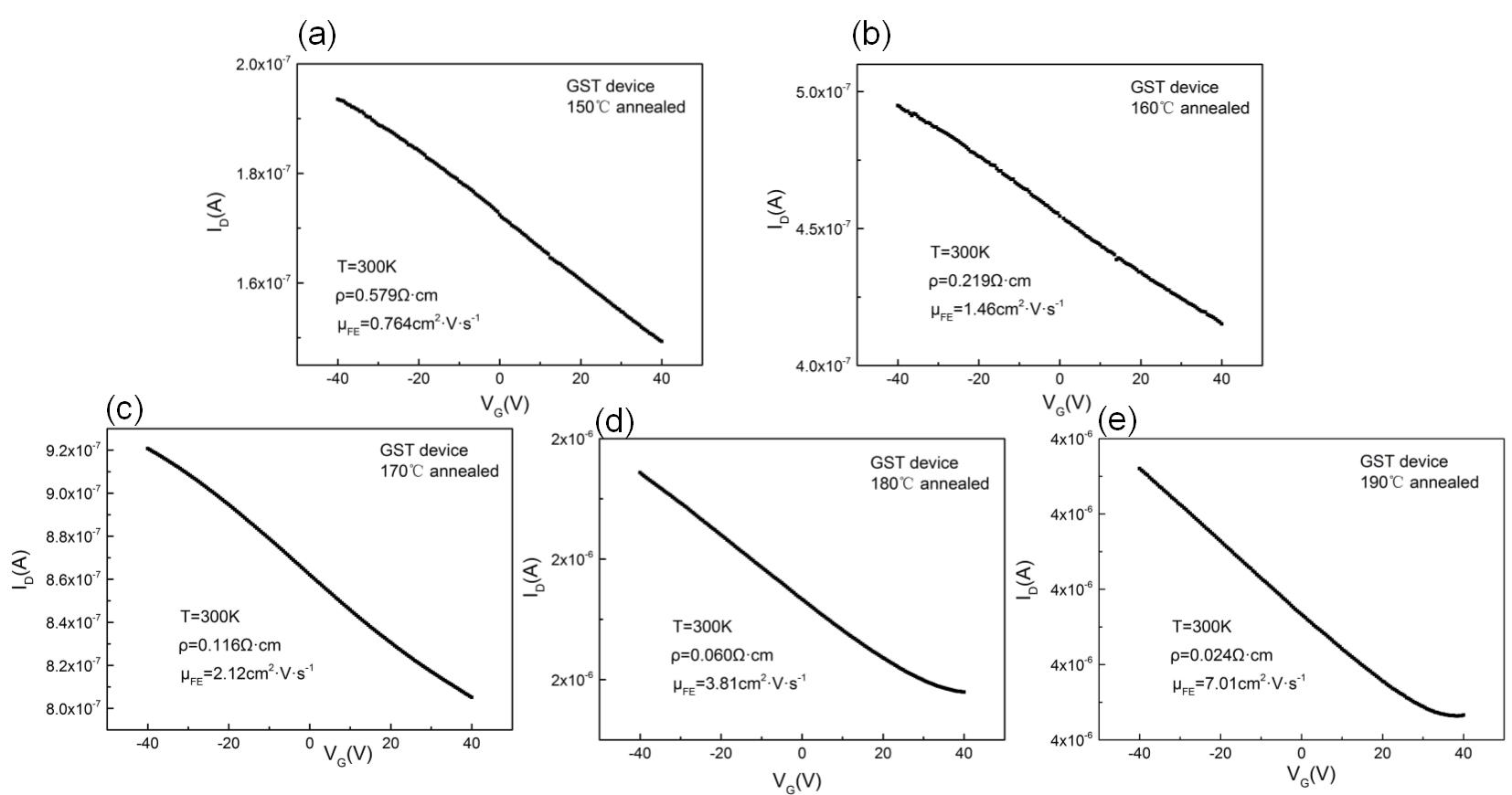
We characterized the device structure by TEM with high resolution and carried out energy spectrum analysis. As shown in the Figure S1(a), the GST film is 10nm and the interface between the GST and other layer (covering layer and dielectric layer) is distinct. From the Figure S1(b), we can confirm material composition of each layer.

Figure S2. Field effect characteristic details of GST device in various annealing temperature.

(a)150 °C (b)160 °C (c)170 °C (d)180 °C (e)190°C

Figure S2 shows the details regarding the field effect experiments. Drain current *I_D_* shows very small and linear dependence on *V_G_*. All measurements were performed in the 300K. The resistivity 𝜌 and field effect mobility 𝜇_FE_ were calculated according to the analysis in the main paper.
